# Validation of the Gravity Model in Predicting the Global Spread of Influenza

**DOI:** 10.3390/ijerph8083134

**Published:** 2011-07-25

**Authors:** Xinhai Li, Huidong Tian, Dejian Lai, Zhibin Zhang

**Affiliations:** 1 Key Laboratory of the Zoological Systematics and Evolution, Institute of Zoology, Chinese Academy of Sciences, 1-5 Beichen West Road, Chaoyang District, Beijing 100101, China; 2 State Key Laboratory of Integrated Pest Management, Institute of Zoology, Chinese Academy of Sciences, 1-5 Beichen West Road, Chaoyang District, Beijing 100101, China; E-Mails: tienhuitung@163.com (H.T.); zhangzb@ioz.ac.cn (Z.Z.); 3 School of Public Health, University of Texas, 1200 Herman Pressler Street, Suite 1006, Houston, TX 77030, USA; E-Mail: dejian.lai@uth.tmc.edu; 4 Faculty of Statistics, Jiangxi University of Finance and Economics, Nanchang 330013, China

**Keywords:** gravity model, influenza A (H1N1), generalized linear model, infectious disease, viral spread

## Abstract

The gravity model is often used in predicting the spread of influenza. We use the data of influenza A (H1N1) to check the model’s performance and validation, in order to determine the scope of its application. In this article, we proposed to model the pattern of global spread of the virus via a few important socio-economic indicators. We applied the epidemic gravity model for modelling the virus spread globally through the estimation of parameters of a generalized linear model. We compiled the daily confirmed cases of influenza A (H1N1) in each country as reported to the WHO and each state in the USA, and established the model to describe the relationship between the confirmed cases and socio-economic factors such as population size, *per capita* gross domestic production (GDP), and the distance between the countries/states and the country where the first confirmed case was reported (*i.e.*, Mexico). The covariates we selected for the model were all statistically significantly associated with the global spread of influenza A (H1N1). However, within the USA, the distance and GDP were not significantly associated with the number of confirmed cases. The combination of the gravity model and generalized linear model provided a quick assessment of pandemic spread globally. The gravity model is valid if the spread period is long enough for estimating the model parameters. Meanwhile, the distance between donor and recipient communities has a good gradient. Besides, the spread should be at the early stage if a single source is taking into account.

## 1. Introduction

Influenza A (H1N1) is one of the most common virus strains causing influenza pandemics in humans [1]. A new strain of influenza A (H1N1) was identified in North America in the spring of 2009. The virus was found easily circulating among humans [2]. Given its highly infectious nature [3] and rapid transmission (made possible via modern transportation [4]), this new influenza had caused a great concern globally [1,5,6]. The World Health Organization (WHO) raised its influenza pandemic threat level to six (the highest level) on 11 June 2009 [2]. On 10 August 2010, WHO announced that the H1N1 influenza virus has moved into the post-pandemic period [7].

During the spread of influenza, spatial waves of infection have been observed between large distant populations [8]. Spatial models of infectious diseases are being used with increasing frequency to characterize these large-scale patterns and to evaluate the impact of interventions [9]. Many models have been developed to study the spatial spread of influenza (e.g., [8,10–13]). Viboud *et al*. [8] proposed a gravity model based on transportation theory, which defines the effects of distance (negative effect) and the size (positive effect) of the ‘donor’ and recipient communities. Compared with multigroup models at the scale of households and workplaces/schools [9], the gravity model is designed for larger spatial scales such as community, city, or country. Following Viboud *et al*.’s study, there is a increasing number of applications of the gravity model in the field of infectious disease spread (e.g., [14,15]) The objective of our analysis is to evaluate at what spatial scale and temporal phase that the gravity model is valid with acceptable model performance. We used influenza A (H1N1) 2009 pandemic as a case study.

## 2. Methods

### 2.1. The Gravity Model

The gravity model considers the effect of distance and the size of the donor and recipient communities [8,16]:

(1) $$C_{ij}=\theta \frac{P_{i}^{\tau_{1}}P_{j}^{\tau_{2}}}{D_{ij}^{\rho }}$$

where *C_ij_* is the disease spread intensity between community *i* (of size *P_i_*) and *j* (of size *P_j_*), *θ*, *τ_1_*, *τ_2_* and *ρ* are parameters to be estimated, and *D_ij_* is the distance between the two communities. In the model, the population sizes are positively related to the intensity and the distance is inversely related. In addition to population size and distance, the economic development level would be another important factor in facilitating physical interaction among people. Therefore, we modified gravity model (1) to the following form:

(2) $$N_{i}=\theta \frac{G_{i}^{w_{1}}P_{i}^{w_{2}}}{D_{i}^{w_{3}}}$$

where *N_i_* is the cases of the influenza A (H1N1) in country *i* (of population *P_i_*), *D_i_* is the distance of country *i* from Mexico, where the first confirmed case was from, *G_i_* is the *GDP* or *GSP per capita. θ*, *w_1_*, *w_2_* and *w_3_* are model parameters all. Although it is not clear where the origin of the influenza A (H1N1) 2009 was precisely, we used the place where the first case was identified (Mexico) as the surrogate for the model. Furthermore, we also applied (2) to establish a statistical relationship between the number of days since 23 April 2009 to the first identified case and these social economic factors.

### 2.2. Model Parameter Estimation and Performance Comparison

We used a generalized linear model (GLM) [17] to estimate model parameters. After log-transformation of the three explanatory variables, the GLM has the form:

(3) $$g(N_{i})=\beta_{0}+\beta_{1}\;\text{ln}(G_{i})+\beta_{2}\;\text{ln}(P_{i})+\beta_{3}\;\text{ln}(D_{i})$$

where the dependent variable *N_i_* was the number of cumulative confirmed cases in a country *i* or state *i*; the independent variables were naturally log-transformed population size *P*, GDP *per capita G*, and distance to Mexico *D*. The number of daily cumulative confirmed cases in all the countries is assumed to be from a negative binomial distribution for both the globe (e.g., for the cases of each country on 6 July 2009, mean = 454.5 < standard deviation = 2644.4) and USA (e.g., for the cases of each state on 24 July 2009, mean = 856.7 < standard deviation = 1295.7). Consequently, we determined the dependant variable (daily confirmed cumulative cases) to follow a negative binomial distribution in the GLM. The link function *g()* is the natural logarithm. The intercept and coefficients of the GLM, *β_0_*, *β_1_*, *β_2_*, and *β_3_*, are identical to parameters *ln(θ)*, *w_1_*, *w_2_*, and *w_3_* respectively in the gravity model (2).

We compared the performance of the gravity model at two spatial scales: global spread and national spread in the USA, assuming a single source of the virus, *i.e*., Mexico. We also compared the model performance at a series of temporal phases: from the beginning on April 24 to July (the last days the data were released for global spread and national spread of Influenza A (H1N1)). The model performance was checked using the P values of each independent variable and the deviance of the generalized linear models, calculated using statistical software R (package “MASS”, function “glm.nb”) [18].

### 2.3. Data Sources

We downloaded *per capita* GDP and population size data of each country for 2009 from the International Monetary Fund (IMF) World Economic Outlook Databases updated on 22 April 2009 (http://www.imf.org/external/ns/cs.aspx?id=28). *Per capita* real GDP of each state in the U.S. for 2009 was downloaded from the website of the U.S. Department of Commerce (http://www.bea.gov/regional/gsp/) updated on 24 November 2010. The population data for each state in the U.S. was obtained from the U.S. Census Bureau (http://www.census.gov/popest/states/NST-ann-est.html). In total, we have records of 168 countries and 50 states (and District of Columbia) in the U.S. The confirmed cumulative cases of influenza A (H1N1) for each country were obtained from the WHO (http://www.who.int/en/) for the period from 23April to 6 July 2009 (the last day that WHO published confirmed cases of influenza A (H1N1) for each country). The confirmed cumulative human cases for each state of the USA were obtained from the Center for Disease Control and Prevention (CDC) website (http://www.cdc.gov/h1n1flu/) for the period from 24 April to 24 July 2009 (the last day that CDC published confirmed cases of influenza A (H1N1) for each state). We used the package “argosfilter” in the software R [18] to calculate the distances between centroids of countries and Mexico, and centroids between states (USA) and Mexico, where the function “distance” was used and the distances were calculated using spherical trigonometry. The centroids of countries and states were calculated using ArcGIS 9.2 [19].

## 3. Results

**Figure 1.**
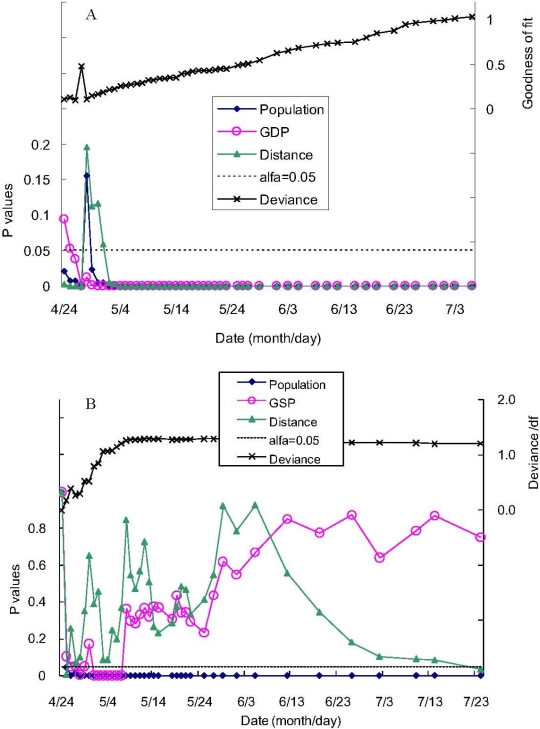
The p-values for testing the significance of the covariates (log-transformed population size (*P*), GDP or GSP (*G*) and distance to each region from Mexico (*D*)) in the GLM with the daily confirmed cumulative human cases of A (H1N1) virus (*N*) as the dependent variable from April 24 to 6 July 2009 (24 July for the USA). A. Global spread model. B. National spread model for the United States of America. The generalized linear model is:
$$g(N_{i})=\beta_{0}+\beta_{1}\;\text{ln}(G_{i})+\beta_{2}\;\text{ln}(P_{i})+\beta_{3}\;\text{ln}(D_{i})$$

The GLM demonstrated that, in log-scale, the number of daily cumulative confirmed cases of influenza A (H1N1) was statistically significantly associated (positively) with population size, except for 28 April and *per capita* GDP, except for 23–25 April, and negatively associated with distance from Mexico, except for 28 April–1 May (Figure 1A). The daily cumulative confirmed cases of influenza A (H1N1) in each state of the USA was positively associated with population size, except for 23 and 24 April, positively associated with *per capita* GSP for a few days only, and not significantly associated with distance to Mexico, except for 25 April (Figure 1B). With additional data [the cases of influenza A (H1N1) accumulated every day], the goodness of fit increased as indicated by the deviance/(degree of freedom) approaching unity (Figure 1). Since May 2009 the patterns were clear that population, GDP, and distance had significant associations with cases of influenza A (H1N1) globally, while only population had a significant association with the influenza cases in each state of the USA (Figure 1). In conclusion, the epidemic gravity model was appropriate for estimating the global spread of influenza A (H1N1), but not for the national spread in the USA.

Using the regressed coefficients of GLM for the day of 6 July 2009, we obtained the gravity model to estimate cases N of influenza A (H1N1) in each country i (omitting the error terms):

(4) $$N_{i}=\frac{G_{i}^{1.547}P_{i}^{1.575}}{e^{3.44}D_{i}^{2.108}}$$

**Figure 2.**
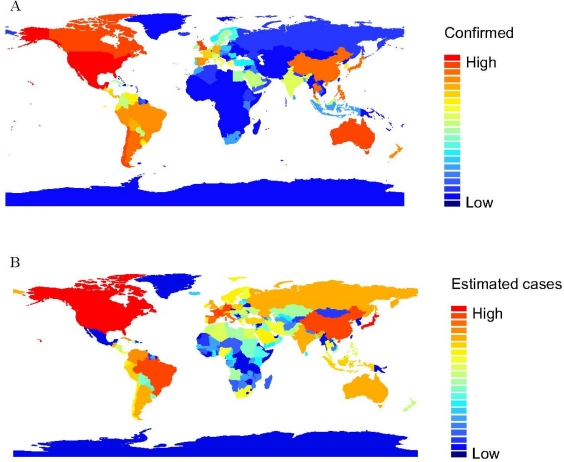
The observed (**A**) and estimated (**B**) values of cumulative confirmed cases of influenza A (H1N1) in each region by the end of the data (6 July 2009) used in this study. The estimated values *N* were based on our modified gravity model incorporating three social and economic factors in Equation (4).

The value and standard errors of the model parameters for variables ln(intercept), ln(G), ln(P), and ln(D) are 3.44 ± 1.496, 1.547 ± 0.111, 1.575 ± 0.113, and 2.108 ± 0.233, respectively. Our estimation of the number of confirmed influenza A (H1N1) cases in each country (Figure 2B) was highly correlated with observed cases as of July 6, 2009 (Figure 2A), with the Spearman correlation coefficient being 0.92, p < 0.0001. Regarding to the data (accumulated confirmed cases of each country on 6 July 2009), 84.9% of its sum of square variance is explained by a simple linear regression (regression of observed cases with the estimated cases) using the ordinary least square method. The estimated values are more homogeneous among countries than the observed cases reported by WHO (Figure 2B).

**Figure 3.**
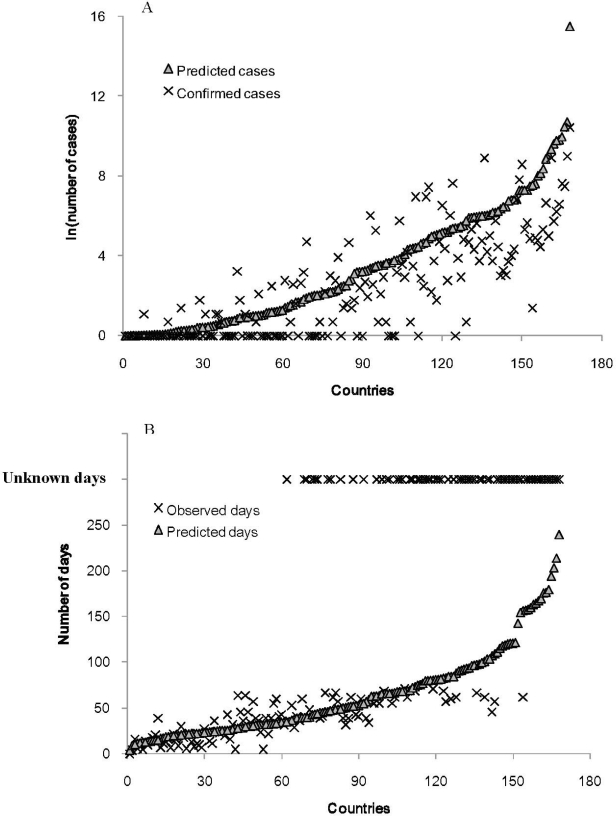
(**A**) The comparison of the number of estimated cases and confirmed cases of influenza A H1N1 for all countries (168 countries in this analysis) on the basis of the data on 6 July 2009. (**B**) The comparison of the number of days (estimated vs. observed) of first infection after 23 April 2009 for all the countries (within the 168 countries, 66 countries had missing values).

For each country, we compared the number of predicted cases from the model and reported confirmed cases based on the data on 6 July 2009 (Figure 3A). Since the number of cases had very high variance, we conducted log transformation to shrink the scale. Using a simple linear regression, we found the predicted values captured 66.78% variance (indicated by R square value) of the number of confirmed cases.

When we used the number of days since 23 April 2009 to the first confirmed infection for each country as the dependent variable in equation (2), we obtained the following:

(5) $$N_{i}=\frac{e^{5.317}D_{i}^{0.486}}{G_{i}^{0.37}P_{i}^{0.285}}$$

We compared the number of predicted days and observed days (Figure 3B). There were 66 countries or regions that had no confirmed cases were treated as missing (Figure 3B). Note that, the coefficients in model (3) had opposite signs in this application (5) as compared to the first application (4). That is, statistically, a higher economic activity (G_i_) and larger population size (P_i_) would lead to a shorter waiting time to the first confirmed case and longer distance (D_i_) would lead to a longer waiting time.

## 4. Discussion

Our results showed that the spread of influenza A (H1N1) among countries was significantly associated to covariates of a set of important socio-economic indicators. The results were consistent with previous findings that air and surface transportation played a significant role in the spread of influenza under both epidemiological survey (e.g., [3]), mathematical epidemic models [4] and theoretical simulations (e.g., [11,13,20]).

We modified the epidemic gravity model with the assumption of a surrogate origin (*i.e.*, Mexico) where the first identified case was from. Although the precise location of the origin of the influenza A (H1N1) 2009 remains unknown, it was believed the virus emerged in Mexico in February 2009 [21]. From May to July 2009, many cases of influenza A (H1N1) in many countries were imported from USA. Because Mexico and USA is close to each other, so that it did not affect the values of distance (the variable used in GLM) very much.

The significance of each covariate (*i.e*., population, GDP, and distance) and model performance varied in the first few days because of small sample sizes (only a few countries and states had identified cases in the early stage of intensive surveillance), and the model became more stable later (Figure 1). Our modified gravity model was not appropriate in modelling the national dynamic of the confirmed cases in the USA (both distance and GSP were not statistically significant). The reasons are: (1) the distances from different states in USA to Mexico were not well ranked, and distance itself is not a good indicator of human mobility here; (2) the spread of the influenza in USA during May and June were not at the early stage of the spread, the inter-states and intra-states spread ware dominant. As a result, we conclude that the gravity model can be applied for influenza spread on the following conditions: (1) the spread period is long enough for estimating the model parameters; (2). the distance between donor and recipient communities has a good gradient; (3) the spread of influenza is at the early stage of if a single source is taking into account.

The daily cumulative confirmed cases of influenza A (H1N1) was used in our analysis, but these cases may not represent the true prevalence of the infection in each region. The number of cases identified was clearly related to the effort and the resources devoted by the health agencies in a country. For a new infectious disease, it is very likely that many cases probably existed already in many parts of the world before the identification of the first case. This is especially true due to the modern transportation systems and possibly many symptomatic and asymptomatic carriers have travelled to many places outside the borders already before the identification of the cases. Following the extensive media reports right after the first identification of the new subtype of the virus, many countries had increased the screening on border-crossing population without paying much attention to their domestic populations at the beginning of the new influenza A (H1N1) 2009 surveillance. The effort of screening only symptomatic cases or their close contacts of confirmed cases entering the country would result finding the cases from a small and biased sample [22].

The three covariates in the model were selected the availability and their important roles in global social and economic interactions. GDP represents the economic activity of the people (for international travel), population size represents the susceptible, and distance represents a possible barrier to infection. Our GLM model provides a quantitative method to estimating the parameters in the model. The model we used was heuristic through conceptual reasoning, but the method of finding the parameters in the model was based on statistical estimation. Mathematical and statistical modelling is an important aspect in addressing public health challenges [23]. Our modelling utilizes social and economic factors and would provide quick insights in understanding the global viral transmission and heath authorities’ efforts.
